# Intact fibroblast growth factor 23 in heart failure with reduced and mildly reduced ejection fraction

**DOI:** 10.1186/s12872-023-03441-2

**Published:** 2023-09-01

**Authors:** Giuseppe Vergaro, Annamaria Del Franco, Alberto Aimo, Francesco Gentile, Vincenzo Castiglione, Federica Saponaro, Silvia Masotti, Concetta Prontera, Niccolò Fusari, Michele Emdin, Claudio Passino

**Affiliations:** 1 Division of Cardiology and Cardiovascular Medicine, Fondazione Toscana Gabriele Monasterio, Via Moruzzi, 1, Pisa, 56127 Italy; 2 Health Science Interdisciplinary Center , Scuola Superiore Sant’Anna, Pisa, Italy; 3 Department of Pathology, University of Pisa, Pisa, Italy

**Keywords:** Fibroblast growth factor, Heart failure, Calcium-phosphate metabolism, Prognosis

## Abstract

**Background:**

Fibroblast growth factor-23 (FGF23) has been associated to left ventricular (LV) hypertrophy and heart failure (HF) severity. We aimed to investigate the clinical correlates and prognostic value of intact FGF23 (iFGF23) in HF patients.

**Methods:**

Patients with stable HF and left ventricular ejection fraction (LVEF) < 50% were prospectively enrolled, managed according to current recommendations and followed over time. iFGF23 was measured at baseline with a fully automated immuno-chemiluminescent assay.

**Results:**

We enrolled 150 patients (82% males; median age 65 years). First, second, and third iFGF23 tertiles were < 35.2 pg/mL, 35.2–50.9 pg/mL, and > 50.9 pg/mL. LVEF decreased from the first iFGF23 tertile to the third tertile (p = 0.014). N-terminal pro-B-type natriuretic peptide (NT-proBNP) increased from the first to the third tertile (p = 0.001), while peak oxygen consumption decreased (p < 0.001). Thirty-five patients (23%) experienced the primary endpoint (all-cause death or HF hospitalization at 5 years), and 26 (17%) the secondary endpoint (all-cause death at 5 years). On multivariable analysis, iFGF23 independently predicted the primary endpoint on top of age, gender and LVEF (HR 4.6 [95% CI 2.1–10.3], p < 0.001), age, gender and eGFR (HR 4.1 [95% CI 1.6–10.3], p = 0.003), as well as age, gender and NT-proBNP (HR 3.6 [95% CI 1.6–8.2], p = 0.002). iFGF23 even reclassified patient risk on top of all the 3 models, with NRI values of 0.65 (95% CI 0.30–1.01), 0.55 (95% CI 0.25–0.88), and 0.60 (95% CI 0.24–0.96), respectively (both p < 0.001).

**Conclusions:**

Circulating iFGF23 is associated with disease severity and outcome in HF patients with reduced and mildly reduced ejection fraction.

**Supplementary Information:**

The online version contains supplementary material available at 10.1186/s12872-023-03441-2.

## Background

Fibroblast growth factor 23 (FGF23) is a peptide hormone mainly synthetized in the bone, regulating phosphate balance by blocking renal tubular phosphate reabsorption and inhibiting the synthesis of 1,25-dihydroxyvitamin D [1]. Moreover, FGF23 has been shown to have pleiotropic effects beyond the regulation of calcium-phosphate homeostasis [2]. FGF23 receptors are indeed expressed in the heart, and several experimental and clinical studies support a role of FGF23 in the development of left ventricular (LV) hypertrophy, fibrosis, and dysfunction [3–6] in different settings, including chronic kidney disease, mostly through an interplay with the renin-angiotensin-aldosterone system (RAAS) [3]. The combination of increased FGF23 and low Klotho – a FGF23 cofactor – levels is associated with a higher risk of cardiovascular death or heart failure (HF) hospitalization in subjects with stable ischemic heart disease. Similarly, the elevation of FGF23 is associated with a significantly increased risk of incident HF in hypertensive populations [7, 8].

Over the past years, FGF23 has emerged as a possible HF biomarker [9]. For example, in the BIOlogy Study to TAilored Treatment in Chronic HF (BIOSTAT-CHF) study, the elevation of FGF23 identified a subset of HF patients with more severe disease and is associated to impaired up-titration of angiotensin-converting enzyme inhibitors (ACEi) and angiotensin receptor blockers (ARBs) [10]. While previous studies had shown that FGF23 is independently associated with cardiovascular mortality and with the composite endpoint of death or heart transplantation in patients with systolic HF [9, 11], a recent *post-hoc* analysis of the Trial of Intensified vs. Standard Medical Therapy in Elderly Patients With Congestive Heart Failure (TIME-CHF) has questioned the predictive power of FGF23 [12].

Most of the studies published so far have reported the clinical value of FGF23 tested with enzyme-linked immunosorbent assays directed to either C-terminal FGF23 (cFGF23). A novel, fully automated FGF23 assay measuring selectively the biologically active hormone, the intact FGF23 (iFGF23), has become available [13], but the clinical significance of iFGF23 in patients with HF remains to be elucidated.

In this study, we aimed to assess correlates and prognostic value of iFGF23, tested with an automated assay, in a fully characterized cohort of patients with systolic HF.

## Methods

### Study population

We enrolled 150 consecutive patients with systolic HF (left ventricular ejection fraction - LVEF < 50%), in a tertiary referral centre for HF (Fondazione Toscana Gabriele Monasterio, Pisa, Italy), between June 2015 and December 2016. HF was diagnosed according to contemporary guidelines [14]. All patients were on stable guideline-recommended HF therapy since at least 3 months. Exclusion criteria were acute coronary syndrome, HF decompensation, coronary artery revascularization or cardiac resynchronization therapy within 3 months before enrolment. Patients underwent 12-lead electrocardiogram, laboratory characterization, echocardiography and cardiopulmonary exercise testing (CPET), over a period of one week. Standard 2D echocardiography was performed and interpreted according to current recommendations [15].

### Laboratory assays

Blood samples were drawn after an overnight fasting period and a 20-minute supine rest [16], and then stored at -80 °C until assays were performed. Plasma renin activity (PRA) and aldosterone were measured using a radioimmunoassay method (DiaSorin S.r.l., Saluggia, Italy) [17, 18]. Plasma epinephrine and norepinephrine were evaluated by means of high-performance liquid chromatography technique using the electrochemical detector CLC 100 (Chromsystems GmbH, München, Germany) [19]. N-terminal fraction of pro-B-type natriuretic peptide (NT-proBNP) was measured with an electrochemiluminescence immunoassay monoclonal method using the Cobas e411 platform (Roche Diagnostics Italia, Monza, Italy) [20]. 25 hydroxy-vitamin D3 (25OHD) was measured by isotope dilution (HPLC-MS/MS), by using the MSMS vitamin D Kit from PerkinElmer (Waltham, MA, USA). Agilent 1290 Infinity UHPLC system was used (Santa Clara, CA, USA), including autosampler, binarypump, and column oven, coupled to an AB Sciex API 4000 triple quadrupole mass spectrometer (Concord, ON, Canada), equipped with an APCI source. Chromatography was performed by a PerkinElmer Brownlee Supra C18 3 μm, 50 × 2.1 mm HPLC column, protected by a PerkinElmer Brownlee Supra C18 Guard Column. Plasma parathyroid hormone (PTH) was measured by the third-generation assay 1–84 PTH assay, chemiluminescent immunoassay (DiaSorin, Saluggia, Italy). Finally, for FGF23 evaluation, an automated iFGF23 immuno-chemiluminescent sandwich assay (DiaSorin, Saluggia, Italy; limit of detection < 10 ng/L, intra-assay coefficient of variation [CV] < 3%, inter-assay CV < 7%) was employed [13].

Estimated glomerular filtration rate (eGFR) was calculated with the Chronic Kidney Disease Epidemiology Collaboration (CKD-EPI) formula.

### Survival analysis and endpoints

Patients underwent follow-up visits every 6 months or as clinically indicated. In December 2022, two independent interviewers retrieved data from the electronic health records and administrative data and through phone calls to the patients, relatives, or general practitioners. The primary endpoint was all-cause death or HF hospitalization at 5 years, and the secondary endpoint was all-cause death at 5 years.

### Statistical analysis

Statistical analysis was performed using SPSS (IBM Statistics, version 26.0, 2019) and R (version 4.2.1, 2022). Normal distribution was assessed through the Shapiro-Wilk test. Normally distributed variables were reported as mean ± standard deviation while non-normally distributed variables as median and interquartile range (IQR). Categorical data were reported as absolute numbers and percentages. According to variable distribution, ANOVA or Kruskal-Wallis test were used for multiple comparisons among groups. χ^2^ or Fisher test were adopted for qualitative variables. Predictors of iFGF23 levels were searched among all baseline characteristics (see Table 1). Ln-transformation was used for all skewed variables; univariate with p < 0.10 were included in a multivariate model. Multicollinearity was excluded by calculating the variance inflation factor (cut-off < 10).

**Table 1:** Baseline characteristics of the overall population and by tertiles of intact fibroblast growth factor 23 (iFGF23).

	Overall (n = 150)	iFGF23 < 35.2 pg/mL (n = 50)	iFGF23 35.2–50.9 pg/mL (n = 50)	iFGF23 > 50.9 pg/mL (n = 50)	p value (trend)
Age, years	65 (54–74)	63 (54–72)	68 (54–76)	67 (57–75)	0.325
Male, n (%)	123 (82)	39 (78)	43 (86)	41 (82)	0.501
BMI, kg/m^2^	26.2 (23.9–29.8)	26.0 (23.6–29.1)	26.2 (24.5–26.7)	26.8 (23.0-31.5)	0.844
NYHA class					
I	39 (26)	17 (34)	13 (26)	9 (18)	0.152
II-III	111 (74)	33 (66)	37 (74)	41 (82)	
Atrial fibrillation, n (%)	21 (28)	5 (10)	12 (24)	4 (8)	**0.036**
Peak VO_2_/kg, mL/(kg·min)	13.6 (10.8–18.0)	17.1 (12.4–21.3)	14.2 (11.1–18.4)	12.3 (10.0-13.9)	**< 0.001**
LVEF, %	32 (27–38)	35 (30–39)	35 (28–40)	30 (25–38)	**0.014**
LVMI, g/m^2^	129 (112–157)	123 (109–143)	132 (120–164)	137 (115–178)	0.103
PAPs, mmHg	35 (29–40)	32 (29–38)	35 (29–40)	36 (30–43)	0.102
E/e’	12 (8–16)	9 (7–12)	13 (10–16)	13 (7–18)	0.089
Haemoglobin (g/dL)	13.6 (1.5)	13.9 (1.1)	13.4 (1.6)	13.7 (1.7)	0.335
eGFR, mL/min/1.73 m^2^	73 ± 24	86 ± 20	75 ± 20	59 ± 23	**< 0.001**
PTH, pg/mL	19.6 (14.3–27.9)	18.4 (12.3–25.4)	17.8 (13.2–25.1)	22.1 (16.7–35.0)	**0.003**
Phosphate, mg/dL	3.5 (3.0-4.1)	3.4 (3.0-3.9)	3.5 (3.0–4.0)	3.7 (3.1–4.4)	0.267
Calcium, mg/dL	9.00 (8.75–9.20)	9.00 (8.70–9.23)	8.90 (8.70–9.10)	9.10 (9.00-9.30)	**0.004**
25-hydroxyvitamin D3, ng/mL	16.26 (9.40-24.49)	15.19 (7.60–21.50)	20.53 (13.63–25.97)	15.83 (9.09–25.44)	0.078
NT-proBNP, ng/L	953 (354–2024)	686 (219–1349)	822 (376–1567)	1680 (635–3285)	**0.001**
PRA, ng/mL/h	1.31 (0.30–3.94)	0.71 (0.20–2.40)	1.13 (0.21–3.15)	2.37 (0.75–7.44)	**0.003**
Aldosterone, ng/dL	137.8 (89.4-209.4)	137.8 (98.9-190.7)	105.5 (71.7-179.6)	168.8 (91.4-322.2)	**0.027**
Epinephrine, ng/L	22 (10–52)	18 (10–45)	18 (10–51)	27 (10–60)	0.575
Norepinephrine, ng/L	404 (283–595)	388 (242–621)	377 (241–523)	510 (316–628)	0.126
ACEi, n (%)	92 (61)	33 (66)	30 (60)	29 (58)	0.557
ARBs, n (%)	46 (31)	17 (34)	12 (24)	17 (34)	0.493
Beta blockers, n (%)	130 (87)	43 (86)	44 (88)	43 (86)	0.708
MRAs, n (%)	92 (61)	30 (60)	28 (56)	34 (68)	0.616
Diuretics, n (%)	104 (69)	31 (62)	35 (70)	38 (76)	0.451

Significant p values are highlighted in bold. ACEi, angiotensin-converting-enzyme inhibitor; ARB, angiotensin receptor blocker; BMI, body mass index; eGFR, estimated glomerular filtration rate; LVEF, left ventricular ejection fraction; LVMI, left ventricular mass index; MRA, mineralocorticoid receptor antagonist; NT-proBNP, N-terminal pro B-type natriuretic peptide; PAPs, pulmonary artery systolic pressure; PRA, plasma renin activity; PTH, parathormone

Patient survival across iFGF23 tertiles was evaluated using the Kaplan-Meier method and Log-Rank statistics (Mantel-Cox). All baseline variables were considered as possible predictors of outcome on Cox regression analysis. The independent prognostic value of iFGF23 was assessed on top of 3 models: age, gender and LVEF (Model 1), age, gender and eGFR (Model 2), age, gender and NT-proBNP (Model 3). This analysis was conducted only for the primary endpoint, given the low number of secondary endpoint events. The number of variables in the models was selected based on the rule of one variable per ten events (and approximating 35 to 40). The added value of iFGF23 was assessed also in terms of continuous net reclassification improvement (NRI).

Two-tailed p values ≤ 0.05 were deemed significant.

## Results

### Baseline characteristics and comparison among iFGF23 tertiles

We enrolled 150 consecutive patients with HF and reduced or mildly reduced EF (median LVEF 32%, IQR 27–38%). Median age was 65 years (54–74 years), 82% were males, and 72% were in New York Heart Association (NYHA) class I-II. Renal function was globally preserved (eGFR 73 ± 24 mL/min/1.73 m^2^), and median NT-proBNP was 953 ng/L (354–2024 ng/L) (Table 1).

Median iFGF23 was 41.8 pg/mL (30.6–55.6 pg/mL) and the first, second, and third tertile were < 35.2 pg/mL, 35.2–50.9 pg/mL, and > 50.9 pg/mL, respectively. As reported in Table 1, maximal oxygen consumption (peak VO_2_/kg) and LVEF decreased from the first to the third tertile, while NT-proBNP increased. Even PTH levels increased form the first to the third tertile of iFGF23.

### Clinical and laboratory correlates of iFGF23

Among all baseline variables, only eGFR (β=−0.323, p < 0.001) and LVMI (β = 0.297, p = 0.001) displayed an independent association with iFGF23 levels (Table 2).

**Table 2:** Predictors of intact fibroblast growth factor 23 (iFGF23): linear regression analysis

	Univariate analysis		Multivariate analysis	
	p	Beta	p	Beta
Age	0.126	-0.180		
Gender (male)	0.652	0.053		
BMI	0.472	0.076		
NYHA class	0.786	0.036		
Atrial fibrillation	0.374	-0.089		
Peak VO_2_/kg	**0.008**	-0.362	0.075	-0.115
LVEF	0.722	-0.044		
LVMI	**0.009**	0.320	0.001	0.297
PAPs	0.115	0.189		
E/e’	0.471	-0.085		
Haemoglobin	0.756	-0.062		
eGFR	**< 0.001**	-0.423	< 0.001	-0.323
PTH	0.424	0.223		
Phosphate	**0.054**	0.156	0.105	0.126
Calcium	0.406	0.163		
25-hydroxyvitamin D3	0.528	0.113		
NT-proBNP	0.480	-0.195		
PRA	0.934	-0.016		
Aldosterone	0.319	0.205		
Epinephrine	0.814	-0.019		
Norepinephrine	0.369	0.073		
ACEi	0.502	0.079		
ARBs	0.781	-0.032		
Beta blockers	0.601	-0.043		
MRAs	0.810	0.019		
Diuretics	0.763	0.025		

Continuous variables were ln-transformed for analysis. The multivariable model included univariate predictors with p < 0.10 (highlighted in bold). ACEi, angiotensin-converting-enzyme inhibitor; ARB, angiotensin receptor blocker; BMI, body mass index; eGFR, estimated glomerular filtration rate; LVEF, left ventricular ejection fraction; LVMI, left ventricular mass index; MRA, mineralocorticoid receptor antagonist; NT-proBNP, N-terminal pro B-type natriuretic peptide; PAPs, pulmonary artery systolic pressure; PRA, plasma renin activity; PTH, parathormone

### Prognostic impact of iFGF23 on mortality

**Fig. 1:**
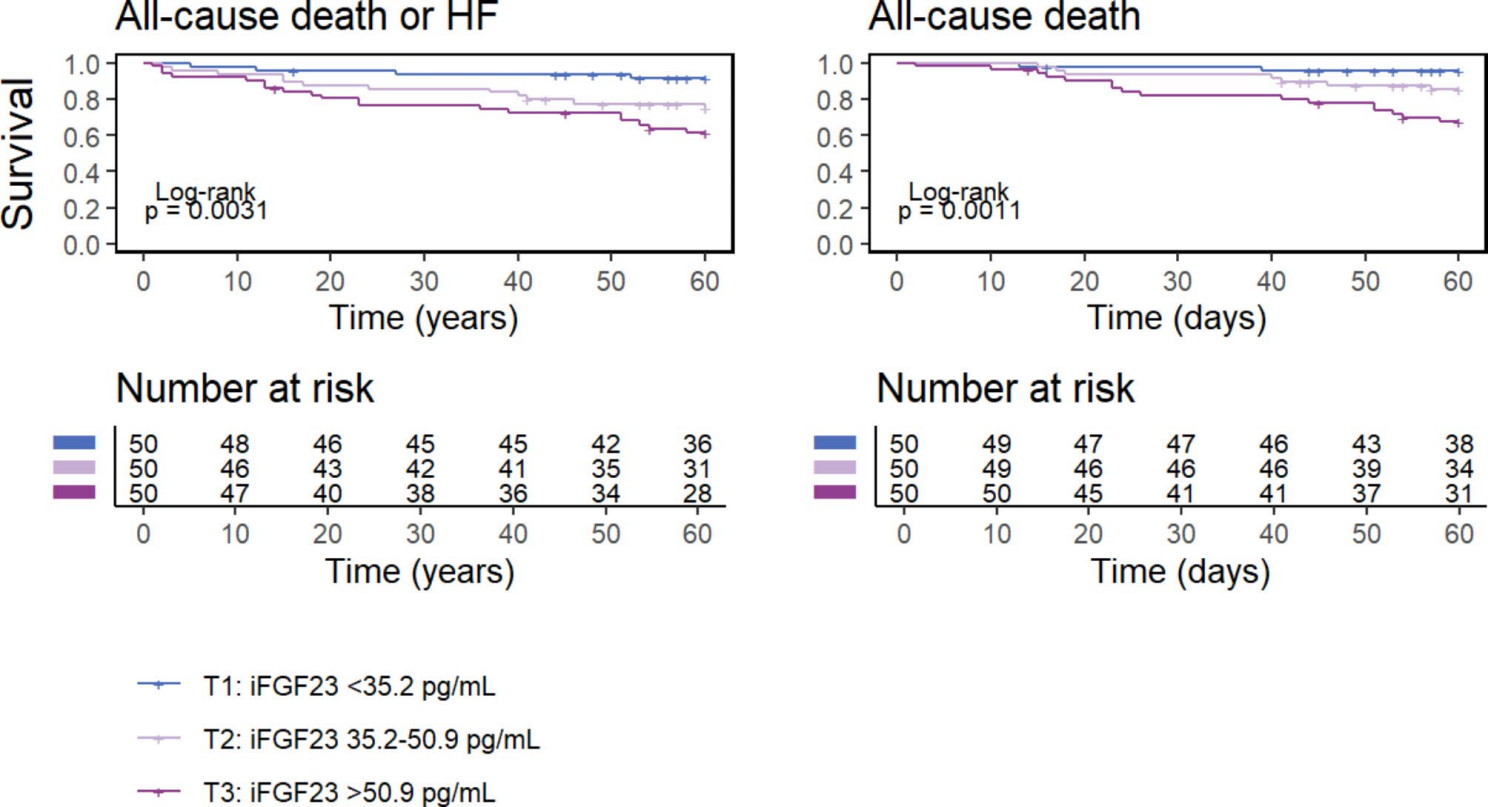
FGF23 tertiles and outcome. Kaplan-Meier curves of 5-year all-cause mortality (a) and of the composite of all-cause mortality and heart failure hospitalizations (b) according to iFGF23 tertiles

The median follow-up period was 4 years (2–5 years). Patients in the third tertile of iFGF23 had a shorter survival free from either the primary or the secondary endpoint (Fig. 1). Thirty-five patients (23%) experienced the primary endpoint (all-cause death or HF hospitalization at 5 years), and 26 (17%) the secondary endpoint of all-cause death. On unadjusted analysis, iFGF23 levels significantly predicted the primary endpoint (hazard ratio [HR] 5.0, 95% CI 2.4–11; p < 0.001) and the secondary endpoint (HR 7.6, 95% CI 3.2–18; p < 0.001). Additional File Table 1 provides the results of the univariate analysis for all the variables listed in Table 1. On multivariable analysis, iFGF23 independently predicted the primary endpoint on top of all the models (see above): Model 1, HR 4.6 [95% CI 2.1–10.3], p < 0.001; Model 2, HR 4.1 [95% CI 1.6–10.3], p = 0.003; Model 3, HR 3.63 [95% CI 1.6–8.2], p = 0.002. iFGF23 even reclassified patient risk on top of all the 3 models, with NRI values of 0.65 (95% CI 0.30–1.01), 0.55 (95% CI 0.25–0.88), and 0.60 (95% CI 0.24–0.96), respectively (both p < 0.001).

## Discussion

**Fig. 2:**
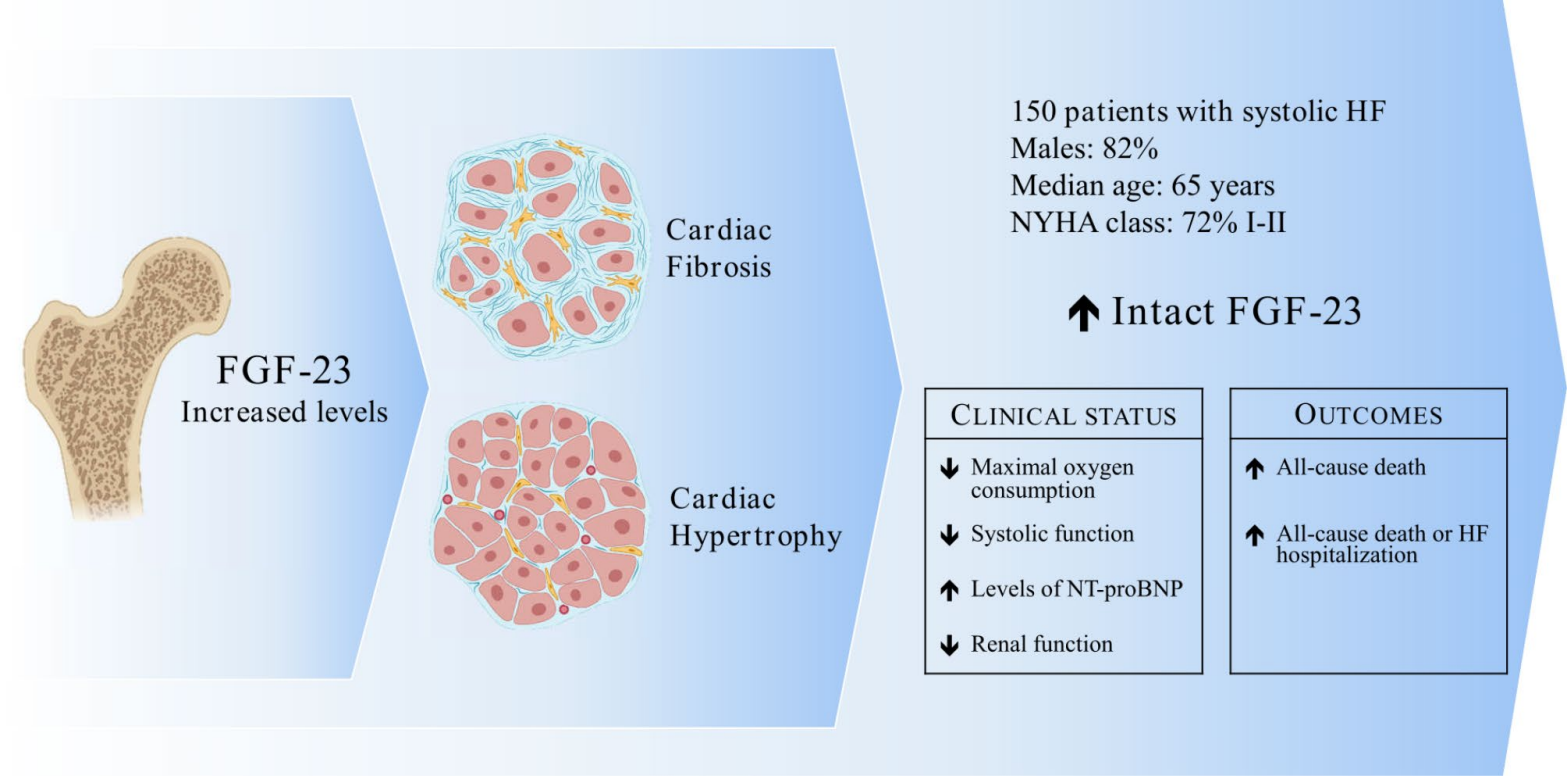
Prognostic impact of FGF23 levels in patients with systolic heart failure (HF). In a cohort of HF patients with systolic dysfunction, higher iFGF23 levels (index of increased cardiac fibrosis and hypertrophy) were associated with clinical severity of the disease, as expressed by lower left ventricular systolic function, higher circulating levels of N- terminal pro-brain natriuretic peptide (NT-proBNP), lower oxygen consumption at cardiopulmonary exercise test and worsening of renal function. Moreover, iFGF23 elevation identified patients at higher risk for all-cause mortality, and a composite endpoint of all-cause death or HF hospitalization

We report that higher iFGF23 levels are associated with greater disease severity and worse outcome in patients with stable HF with reduced or mildly reduced EF (Fig. 2).

FGF23 plays a key role in regulating phosphate homeostasis. Elevated FGF23 levels increase fractional phosphate excretion at kidney level and inhibit PTH synthesis and secretion by parathyroid cells [21]. On the other hand, phosphate itself is a major regulator of FGF23: dietary phosphate loading increases circulating FGF23 levels, whereas phosphate depletion decreases FGF23 expression [22]. The evidence of the independent association between iFGF23 and phosphate in our population is therefore supported by pathophysiological mechanisms of FGF23 regulation.

Renal function, assessed by eGFR, emerged as another independent predictor of iFGF23 in our study. The inverse correlation between eGFR and iFGF23 is likely due to both direct and indirect mechanisms, consisting in the reduced renal excretion of iFGF23 and increased phosphate levels, respectively, following decreasing renal function. Nevertheless, iFGF23 values in our cohort was still in the normal to high-normal range, in line with other studies investigating cardiovascular setting and without severe renal impairment [23], since higher levels are usually confined to individuals with a severe chronic renal dysfunction.

There is experimental evidence that FGF23 may enhance RAAS activity [24], although more recent clinical data are controversial [7]. An analysis from the Multi-Ethnic Study of Atherosclerosis (MESA) study could not demonstrate a relationship between iFGF23 and either circulating aldosterone or PRA in hypertensive patients [7]. Conversely, data from a large population with new onset or worsening HF have shown that aldosterone levels were independently associated with higher FGF23 [10]. This is the first study addressing the possible association between biohumoral indices of RAAS activation and iFGF23 in the setting of chronic HF. In our population, both aldosterone and PRA were associated to iFGF23 circulating levels at univariate analysis, but the association was lost after multivariate adjustment.

Stimulation of RAAS activity has been postulated as one of the mechanisms underlying the association between FGF23 and LV hypertrophy [5]. Available data also suggest that activated RAAS induces myocardial expression of FGF23 [25], that could in turn promote fibrosis-related pathways in fibroblasts and consequently cardiac remodelling and dysfunction [26]. Experimental studies show how transgenic mice with overexpression of a constitutively active FGF receptor-1 develop LV hypertrophy [27]. Further, FGF23 blockade reverses the hypertrophic growth of isolated myocytes in vitro and established LV hypertrophy in vivo [28]. We report here that LVMI is independently associated with iFGF23 in a cohort of stable systolic HF patients. These findings could support the hypothesis that FGF23 is not only a biomarker of increased risk but acts as a direct endocrine and paracrine player in the pathophysiology of HF.

Our findings that iFGF23 is an independent predictor of outcome in patients with HF is in line with previous evidence reporting FGF23 as a correlate of HF-related outcome, in patient with or without kidney disease [7]. Notably, it retained an independent prognostic value on top of age, gender and LVEF, eGFR or NT-proBNP, which are important determinant of outcome in patients with HF. Recently, data from 382 patients enrolled in the TIME-CHF (Trial of Intensified vs. Standard Medical Therapy in Elderly Patients With Congestive Heart Failure) cohort have questioned the role of FGF23 in risk stratification [12]. Still, this analysis included patients with more severe disease (no patient in NYHA class I vs. 26% in our population), and both cFGF23 and iFGF23 were tested with commercially available ELISA assays. We used rather a fully automated immunoassay for iFGF23 that is approved for clinical use and shows excellent analytical characteristics, allowing standard results and references. In addition, this assay measures the intact form of (iFGF23), showing a stronger biological activity than cFGF23 [29]. Furthermore, all blood samples were performed at the same time of the day, to avoid potential circadian variations of FGF23 levels between patients, and testing included a full characterization of RAAS activation as well as of phosphate and calcium metabolism.

FGF23 is increasingly recognized as a marker and a mediator of disease progression in HF. Our findings support the role of iFGF23 as a player in cardiac remodelling following renal dysfunction and a tool for risk stratification in HF patients. Characterization of the clinical significance of iFGF23 is of great relevance given the availability of different pharmacological approaches for FGF23 blockade. These include inhibitors of gastrointestinal sodium/hydrogen exchanger isoform 3 such as tenapanor [30], and cinecalcet, a calcimimetic agent that has been shown to reduce both serum FGF23 levels and rates of cardiovascular death and major cardiovascular events in haemodialysis patients [31]. Finally, burosumab, a monoclonal antibody targeting FGF23, has been recently tested in patients with X-linked hypophosphatemia [32].

Several limitations must be acknowledged. Serial measurements of iFGF23 were not performed, therefore changes in iFGF23 during the follow-up and their influence on patient outcome cannot be assessed. Moreover, we report data from a single tertiary centre and a quite small cohort, which may limit the generalizability of our findings.

In conclusion, circulating iFGF23 is associated with disease severity and outcome in HF patients with reduced and mildly reduced ejection fraction.

### Electronic supplementary material

Below is the link to the electronic supplementary material.

Additional File Table 1: Univariate predictors of outcome

## Data Availability

The datasets generated and/or analysed during the current study are not publicly available due to the ongoing research for other items of interest in the same enlarged population, but are available from the corresponding author on reasonable request.

## Abbreviations

25OHD: 25 hydroxy-vitamin D3
ACEi: angiotensin-converting enzyme inhibitors
ARBs: angiotensin receptor blockers
BMI: body mass index
cFGF23: C-terminal fraction of FGF23
eGFR: estimated glomerular filtration rate
FGF23: fibroblast growth factor-23
HF: heart failure
iFGF23: intact FGF23
LV: left ventricular
LVEF: left ventricular ejection fraction
LVMI: left ventricular mass index
MRAs: mineralocorticoid receptor antagonists
NT-proBNP: N-terminal fraction of pro-B-type natriuretic peptide
NYHA: New York Heart Association
PASP: pulmonary artery systolic pressure
PRA: plasma renin activity
PTH: parathyroid hormone
RAAS: renin-angiotensin-aldosterone system
