# Supplementary material for: Intact fibroblast growth factor 23 in heart failure with reduced and mildly reduced ejection fraction
Source: BMC Cardiovasc Disord. 2023 Sep 1;23:433. doi: 10.1186/s12872-023-03441-2 (PMC10474676; doi:10.1186/s12872-023-03441-2)
Supplement: Supplementary file 1 — Additional File Table 1: Univariate predictors of outcome [file 12872_2023_3441_MOESM1_ESM.docx]

**SUPPLEMENTAL MATERIAL**

**Intact fibroblast growth factor 23**

**in heart failure with reduced and mildly reduced ejection fraction**

Giuseppe Vergaro, MD, PhD,^1,2^ Annamaria Del Franco, MD,PhD,^1,2*^ Alberto Aimo, MD,PhD,^1,2*^ Francesco Gentile, MD,^1^ Vincenzo Castiglione, MD,^1^ Federica Saponaro, MD, PhD,^3^ Silvia Masotti, BSc,^1^ Concetta Prontera, PhD,^1^ Niccolò Fusari, MD,^1^ Michele Emdin, MD, PhD,^1,2^ Claudio Passino, MD^1,2^

1. Division of Cardiology and Cardiovascular Medicine, Fondazione Toscana Gabriele Monasterio, Pisa, Italy

2. Interdisciplinary Center for Health Sciences, Scuola Superiore Sant’Anna, Pisa, Italy

3. Department of Pathology, University of Pisa, Italy

* The Authors contributed equally to the paper.

**Supplemental Table 1. Univariate predictors of outcome.**

|  | **All-cause death or HF hospitalization** | | **All-cause death** | |
| --- | --- | --- | --- | --- |
|  | **HR (95% CI for HR)** | **p** | **HR (95% CI for HR)** | **p** |
| Age | 2.4 (0.54-11) | 0.250 | 2.3 (0.39-13) | 0.360 |
| Gender (male) | 2.5 (0.76-8.2) | 0.130 | 2.6 (0.61-11) | 0.190 |
| BMI | 1.2 (0.18-7.6) | 0.860 | 1 (0.11-9.7) | 0.970 |
| NYHA class | 2.9 (1.7-4.8) | **<0.001** | 2.8 (1.6-5) | **<0.001** |
| Atrial fibrillation | 0.33 (0.079-1.4) | 0.13 | 0.23 (0.032-1.7) | 0.150 |
| Peak VO_2_ | 0.23 (0.1-0.53) | **<0.001** | 0.27 (0.099-0.74) | **0.011** |
| LVEF | 0.22 (0.077-0.63) | **0.005** | 0.16 (0.047-0.53) | **0.003** |
| LVMI | 3.4 (0.96-12) | 0.057 | 3.7 (0.81-17) | 0.092 |
| PAPs | 8.9 (3-26) | **<0.001** | 13 (3.5-44) | **<0.001** |
| E/e’ | 4.1 (1.4-12) | **0.011** | 3.9 (1.1-13) | **0.031** |
| Haemoglobin | 0.86 (0.69-1.1) | 0.16 | 0.82 (0.64-1.1) | 0.130 |
| eGFR | 0.98 (0.96-0.99) | **0.001** | 0.97 (0.95-0.99) | **<0.001** |
| PTH | 3.1 (1.7-5.9) | **<0.001** | 4.8 (2.1-11) | **<0.001** |
| Phosphate | 1.3 (0.33-4.8) | 0.740 | 0.74 (0.15-3.7) | 0.710 |
| Calcium | 0.58 (0.098-3.4) | 0.54 | 1.9 (0.33-11) | 0.470 |
| 25-hydroxyvitamin D3 | 0.68 (0.46-1) | 0.062 | 0.59 (0.38-0.92) | **0.020** |
| NT-proBNP | 1.9 (1.4-2.7) | **<0.001** | 1.9 (1.3-2.9) | **<0.001** |
| PRA | 1.5 (1.2-1.9) | **0.001** | 1.8 (1.3-2.4) | **<0.001** |
| Aldosterone | 1.5 (0.97-2.5) | 0.068 | 1.7 (0.98-3) | 0.057 |
| Epinephrine | 1 (0.68-1.5) | 0.950 | 0.97 (0.6-1.5) | 0.890 |
| Norepinephrine | 1.7 (1-2.9) 4.3 0.038 | **0.038** | 1.9 (1-3.5) | **0.048** |
| ACEi | 0.72 (0.37-1.4) | 0.340 | 0.46 (0.21-1) | 0.055 |
| ARBs | 1 (0.5-2.1) | 0.940 | 1.5 (0.69-3.4) | 0.300 |
| Beta blockers | 0.7 (0.29-1.7) | 0.430 | 1.1 (0.33-3.7) | 0.870 |
| MRAs | 2.5 (1.3-4.8) | **0.007** | 2.2 (1-4.7) | **0.046** |
| Diuretics | 2.2 (0.93-5.4) | **0.071** | 2.3 (0.79-6.7) | 0.130 |

Significant p values are highlighted in bold. ACEi, angiotensin-converting-enzyme inhibitor; ARB, angiotensin receptor blocker; BMI, body mass index; eGFR, estimated glomerular filtration rate; LVEF, left ventricular ejection fraction; LVMI, left ventricular mass index; MRA, mineralocorticoid receptor antagonist; NT-proBNP, N-terminal pro B-type natriuretic peptide; PAPs, pulmonary artery systolic pressure; PRA, plasma renin activity; PTH, parathormone.
